# Impact of environmental factors on diversity of fungi in sediments from the Shenzhen River Estuary

**DOI:** 10.1007/s00203-023-03438-7

**Published:** 2023-02-23

**Authors:** Wenzhen Lin, Xin Liu, Linfeng Gong, Ruzhen Liu, Minghuang Ling, Chiming Guo, Hongyan Meng, Zhuhua Luo, Xiaona Du, Ying Guo, Wei Xu

**Affiliations:** 1 Fujian Key Laboratory of Subtropical Plant Physiology and Biochemistry, Fujian Institute of Subtropical Botany, Xiamen, 361006 China; 2 School of Life Sciences, Anhui Agricultural University, Hefei, 230036 China; 3 Key Laboratory of Marine Biogenetic Resources, Third Institute of Oceanography, Ministry of Natural Resources, 178 Daxue Road, Xiamen, 361005 China; 4 Sanquan College of Xinxiang Medical University, Xinxiang, 453000 Henan China

**Keywords:** Environmental factors, Fungal diversity, Shenzhen River, Estuary

## Abstract

In this study, to explore the relationship between environmental factors and fungal diversity in the Shenzhen River ecosystem, multiple methods including chemical analysis, culture isolation, qPCR analysis of fungal ITS region and ITS-based Illumina next-generation-sequencing were integrated. A total of 115 isolates were finally isolated and could be classified into 23 genera. Top three abundant genera isolated were *Meyerozyma* (18 strains), *Aspergillus* (17 strains) and *Penicillium* (14 strains). Based on the Illumina sequencing approach, 829 OTUs were affiliated to seven phyla, 17 known classes, and 162 genera, indicating the Shenzhen estuary sediments are rich in fungal diversity. The major fungal genera were *Meyerozyma*, *Trichoderma* and *Talaromyces*. Environmental factors showed a gradient change in Shenzhen estuary, and fungal abundance was only significantly correlated with NH_4_^+^. Shannon index was significantly correlated with pH and IC (*P* < 0.05). Principal coordinate analysis based on OTU level grouped into three clusters among sampling sites along with the IC and pH gradient. Functional guilds analysis suggests most of the fungi in this studying area were almost all saprotrophs, suggesting a large number of saprophytic fungi may play a significant role in the organic matter decomposition and nutrient cycling process. In summary, this study will deepen our understanding of fungi community in Shenzhen River ecosystem and their distribution and potential function shaped by environmental factors.

**Supplementary Information:**

The online version contains supplementary material available at 10.1007/s00203-023-03438-7.

## Introduction

In estuary and coastal areas, sediments are one of the most productive environments on earth due to vertical absorption of nutrients and organics matter from the upper water layer, harboring a higher microbial population than the corresponding water bodies in terms of biomass and taxon richness (Whittaker and Likens 1975; Zinger et al. 2011; Liu et al. 2015a; Liu et al. 2015b). The estuary ecosystem, which provides a habitat for different organisms ranging from bacteria, fungi and algae to invertebrates, birds, and mammals, is regarded as the main habitation for various microbial communities due to its excellent biological productivity (Crump et al. 1999; Beck et al. 2001; Lesage et al. 2001; Burton et al. 2010; Wang et al. 2019; Scharler et al. 2020; Sheeba et al. 2020). Salinity as one of the important environmental factors will influence the fungal diversity. And the estuarine habitat is characterized by a salinity gradient. Li et al. proposed that salinity, total nitrogen (TN) and C/N significantly influenced the spatial distribution patterns of fungal communities (Li et al. 2016b). The results of coastal sites off the South China Sea (Pearl River estuary, Shenzhen Bay, and Daya Bay) showed that salinity and nitrate were the major factors driving the variations among fungal communities, and Ascomycota was positively correlated with salinity and negatively correlated with salinity and negative correlated with nitrate and nitrite (Wang et al. 2019).

The shallow estuary sediments which are deeply affected by current of fresh water interaction with sea water and tidal changes. Meanwhile, the salinity and nutrients create a good gradient habitation supporting complicate microbial communities who playing important role in biogeochemical cycling (Baker et al. 2015; Jiang et al. 2019; Park et al. 2020). Most studies focused on microbial assemblages present in a range of coastal habitats such as woody parts of trees, mangrove and estuary sediments (Ramsay et al. 2000; Ramírez-Elías et al. 2014; Yang et al. 2014; Luo et al. 2017; Marcos et al. 2018; Jiang et al. 2019; Li et al. 2019; Wang et al. 2019; Wang et al. 2021; Kalkan and Altuğ 2020).

Fungi are a subset of microeukaryotes, which are an ecologically and functionally diverse kingdom of microbial organisms (Khomich et al. 2017). Recently there has been growing interest in microbial and eukaryotic diversity in different coastal environments, such as tidal marshes (Buchan et al. 2002; Mohamed and Martiny 2011), and mangroves (Arfi et al. 2012), the Arctic (Zhang et al. 2015), the northern Chinese seas (Li et al. 2016a; Li et al. 2016b; Wang et al. 2017) and the coastal beach of China (Wang et al. 2021). Our previous knowledge of marine fungal diversity is mainly based on culture methods, however, the development of high-throughput sequencing technology has allowed the exploration of fungal community with sufficient sequence coverage and enabled robust and comprehensive assessment of fungal distribution pattern. Arfi et al. (2012) investigated the anoxic mangrove sediments of Saint Vincent Bay using 454 pyrosequencing of the nuclear ribosomal internal transcribed spacer and revealed that Agaricomycetes was the dominant fungal class. The most abundant operational taxonomic units (OTUs) were affiliated to the *Sistotremastrum* (Trechisporales), *Dipodascus australiensis* (Saccharomycetales), *Alternaria* (Pleosporales) and unknown Lecanoromycete. Li et al. (2016b) analyzed the fungal community from various intertidal habitats of Chinese seas based on ribosomal RNA (rRNA) internal transcribed spacer region 2 (ITS2) metabarcoding, their results showed that there were 526 genera of fungi. Wang et al. (2018) studied the abundance and diversity of planktonic fungi in the coastal waters of the Bohai Sea, and found that fungi were mainly composed of Ascomycota, Basidiomycota, and Chytridiomycota. The estimated average fungi-specific 18S rRNA gene qPCR abundance varied within 4.28 × 10^6^ and 1.13 × 10^7^copies L^−1^. This result indicated that the Chinese intertidal region has a rich diversity of fungi and a high abundance of fungi (Wang et al. 2018).

Shenzhen River is a tidal river on the border between Hong Kong and Shenzhen, which represents one of the most important and complex ecosystems linking the highly developing land area and the South China Sea. The shallow estuary sediments and their microbial communities are a global hotspot for biogeochemical cycling. Although prokaryotic assemblages in the Shenzhen estuary have been studied (Yang et al. 2014; Zhang et al. 2015; Zhou et al. 2017; Qiu et al. 2019; Wu et al. 2019), fungal research still lacks sufficient knowledge. In this study, based on the ITS rRNA using cultivation-dependent and cultivation-independent methods, we aim to survey the diversity and community compositions of fungi in Shenzhen River Estuary. Furthurly, through quantitative PCR combined with environmental factors, we can address the following questions: which environmental parameters in the Shenzhen River Estuary are critical for maintenance of the community structure?

## Materials and methods

### Sampling, physicochemical parameter measurement, and DNA isolation

**Fig. 1:**
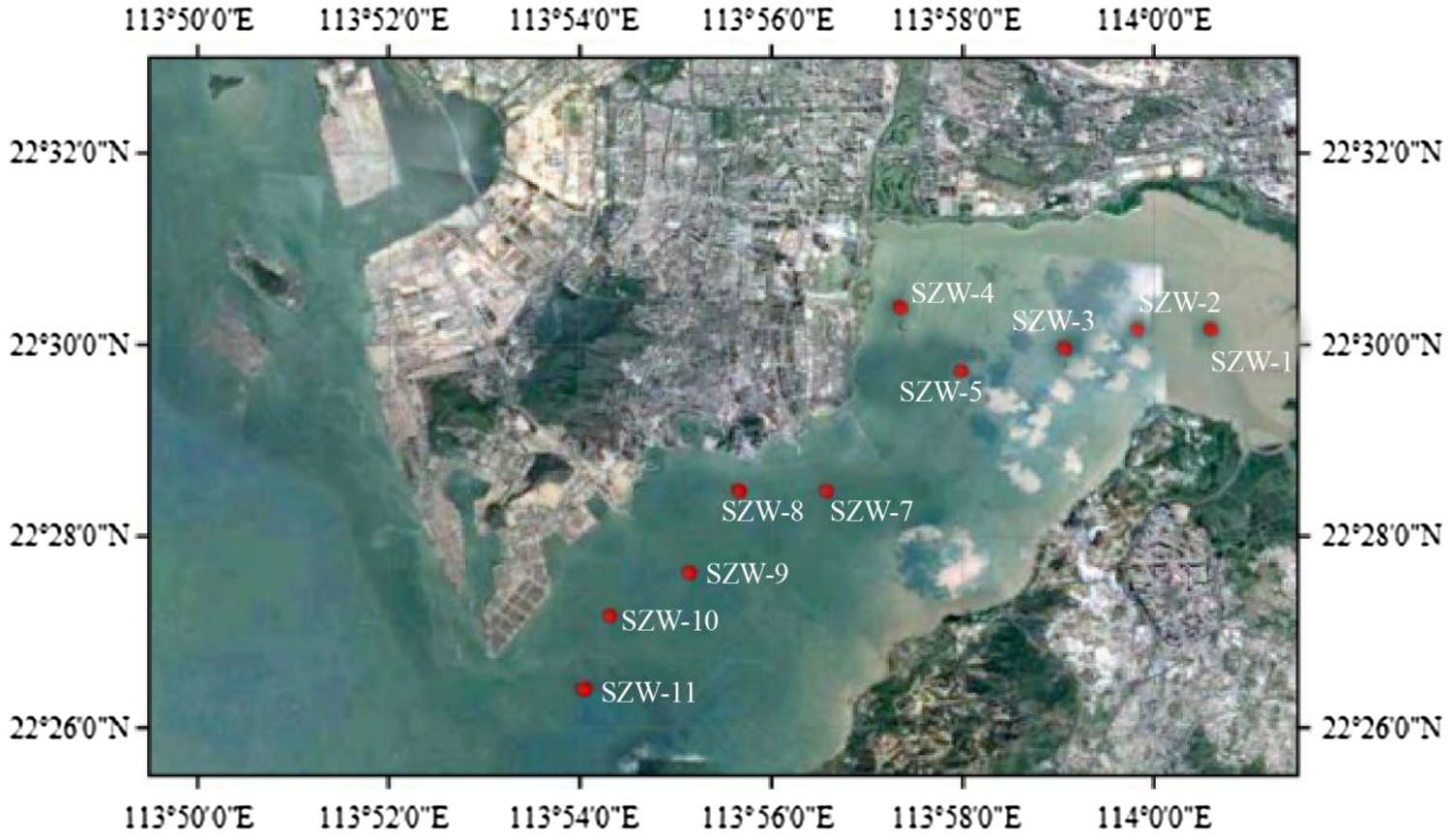
Location of the sediment sampling sites in the Shenzhen River Estuary, China

The layout principle of sampling stations is based on the salinity gradient and the fresh water current to sea water. Sampling was carried out in Apr. 2017 on the edge of the Shenzhen River and estuary of Shenzhen. Surface sediment samples from a depth of 0–5 cm were collected from the river cross-section (water depth ≈ 1–3 m) in an area of 5 × 5 m at the selected sampling sites (Fig. 1). The surface sample collect using GRAB type surface sediment sampler. A total of 10 sediments samples were obtained. All the sediment samples were transferred into sterile plastic bags and placed into iceboxes immediately and transported back to the laboratory shortly after collection. The samples used for molecular studies were stored at − 80 °C, and the samples used for physicochemical analysis were immediately processed. After transportation to the laboratory, a part of the sediments was sub-packaged into 50 mL sterile tubes and stored at − 20 °C for DNA extraction before sequencing, and the remaining samples were stored at 4 °C before the analysis of physicochemical properties. Temperature, salinity and pH were measured simultaneously with sampling, and the remaining environmental factors were measured later.

The physicochemical parameters, including NH_4_^+^, NO_2_^−^, NO_3_^−^, total nitrogen (TN), total carbon (TC), total organic carbon (TOC), and inorganic carbon (IC) were measured as previously reported (Zhou et al. 2016). The results are shown in Table 1. For DNA extracting, 0.5 g of wet sediments from each sample were used for DNA extraction by Power Soil^®^ DNA Isolation Kit (MO BIO, Carlsbad, CA, USA) according to the instructions.

**Table 1:** Details and physicochemical characteristics of the sediment samples collected from the Shenzhen River Estuary

Sample	Longitude	Latitude	Depth (m)	pH	Salinity (ppt)	Temperature (℃)	NO2^−^ (mg N/kg)
SZW-1	114°0′34′′	22°30′9′′	1.4	7.16	17.02	19.523	0.01
SZW-2	113°59′49′′	22°30′7′′	1.2	7.02	21.24	19.292	0.01
SZW-3	113°59′03′′	22°29′59′′	1.7	7.13	22.93	19.351	0.011
SZW-4	113°57′20′′	22°30′24′′	2.8	7.31	24.54	19.421	0.022
SZW-5	113°57′57′′	22°29′43′′	3.5	7.31	25.65	19.319	0.023
SZW-7	113°56′34′′	22°28′28′′	5.7	7.08	26.52	18.99	0.01
SZW-8	113°55′40′′	22°28′28′′	3.8	7.47	26.93	18.394	0.036
SZW-9	113°55′10′′	22°27′38′′	5	7.41	26.41	18.333	0.039
SZW-10	113°54′24′′	22°27′12′′	5.3	7.3	27.21	18.456	0.016
SZW-11	113°54′8′′	22°26′28′′	5.5	7.28	28.07	18.75	0.012

Sample	NO3^−^ (mg N/kg)	NH4^+^ (mg N/kg)	TN^a^ (mg N/kg)	IC^b^ (mg C/kg)	TOC^c^ (mg C/kg)	TC^d^ (mg C/kg)
SZW-1	0.599	30.611	1361	11.44	2069	2080
SZW-2	0.547	41.698	1249	16.86	2012	2029
SZW-3	0.74	40.659	1354	16.53	2615	2631
SZW-4	0.614	33.404	1312	12.42	2176	2189
SZW-5	0.456	22.072	955.1	14.46	1662	1676
SZW-7	0.785	30.219	1117	13.06	2094	2107
SZW-8	0.534	12.923	884.1	14.16	1539	1553
SZW-9	0.685	20.579	1020	14.66	1441	1455
SZW-10	0.565	15.105	847.6	12.83	1449	1462
SZW-11	0.499	12.43	647.9	12.58	1112	1125

^a^TN (total nitrogen)

^b^IC (inorganic carbon)

^c^TOC (total organic carbon)

^d^TC (total carbon)

### Isolation and identification of fungal isolates

Isolation of fungi by dilution coating plate method with six different media was carried out as previously reported (Xu et al. 2017). Under sterile conditions, burning heat sterilized spoon was used to dispense the sediment into a 15 mL sterile conical tube with 1 g sediment per tube. First, 9 mL of sterilized artificial seawater was added to the conical tube and shaken at 180 rpm for 20 min. Second, sterile artificial seawater was used to dilute the suspension solution into 10^–1^, 10^–2^ times dilution and the tube was shaken at 135 rpm for 20 min to mix the sediment in the tube. Third, under sterile conditions, we used a pipette to draw 200 μL of the sample solution from the six kinds of culture medium (Xu et al. 2017) and plated evenly on the culture medium, and sealed with a parafilm and then incubate with temperature 25℃ from 7 to 14 days until the clone appeared. Every experiment was conducted in triplicate. All pure fungal cultures with a distinct colony morphology were maintained on corn meal agar (CMA) plates. The DNA extract from the fungal isolates using FastDNA spin kit for soil kit as the cell wall are hard to destroy by classical microbial DNA isolate kit. Fungal isolates were identified by ITS rDNA sequences using ITS5 (5′-GGAAGTAAAAGTCGTAAACAAGG-3′) and ITS4 (5′-TCCTCCGCTTATTGATATGC-3′) (White et al. 1990). PCR reaction was performed at the conditions according to Li’s report (Li et al. 2018). Related PCR reagents were from Invitrogen, USA. The PCR products were purified using a Gel Extraction Kit (Tiangen Co., Beijing, China) and the sequencing progress was performed by Majorbio (China) using ABI 3730XL sequencer (ABI, USA).

Based on the full length of the sequences, phylogenetic relationships were inferred using the Neighbor-Joining method (Saitou and Nei 1987). Bootstrap values based on 1000 random replicates are shown next to the branches (Felsenstein 1985). The evolutionary distances were computed using the Kimura’s 2-parameter method (Kimura 1980) in the units of the number of base substitutions per site. This analysis involved 373 nucleotide sequences including 46 new sequences in this study and reference sequences downloaded from NCBI. All ambiguous positions were removed from each sequence pair (pairwise deletion option). There were a total of 302 positions in the final dataset. Evolutionary analyses were conducted using MEGA X (Kumar et al. 2018). The taxonomy classification were based on the ITS phylogenetic tree according to the reference sequences’ taxon name.

### PCR amplification and illumina sequencing

The soil DNA was extracted from all the samples within 24 h of sampling and stored at − 80 °C. The ITS region of the fungal rRNA gene was amplified by PCR using primers ITS3_KYO2F (5′-GATGAAGAACGYAGYRAA-3′) and ITS4R (5′-TCCTCCGCTTATTGATATGC-3′) (Toju et al. 2012). PCR reaction conditions were adapted from Sun’s report (Sun et al. 2019).

PCR products were extracted from 1% agarose gels and purified by the AxyPrep DNA Gel Extraction Kit (Axygen Biosciences, Union City, CA, USA) and quantified using QuantiFluor -ST (Promega, US). Sequencing libraries were generated using the NEB Next R UltraTM DNA Library Prep Kit for Illumina (NEB, USA) according to the manufacturer’s recommendations, and index codes were added. The library quality was assessed using the QuantiFluorTM-ST Blue fluorescence quantitative system (Promega, USA). The raw reads were uploaded into the NCBI Sequence Read Archive (SRA) database (Accession Number: SAMN12669234-SAMN12669243).

### Quality control and reads assembly

Raw sequencing data includes sequence domains of adapters and low-quality bases which would affect sequence assembly and analysis. To get high quality clean reads, raw reads were further filtered according to the previously reported rules (Lu et al. 2014; Li et al. 2017; Xu et al. 2017). Paired-end clean reads were merged as raw tags using FLASH (v 1.2.11) (Tanja and Steven 2011), and then, noisy sequences of raw tags were filtered using QIIME (V1.9.1) (Caporaso et al. 2010) pipeline under specific filtering conditions to obtain the high-quality clean tags (Bokulich et al. 2013). Clean tags were searched against the reference database (http://drive5.com/uchime/uchime_download.html) to perform reference-based chimera checking using UCHIME algorithm (Edgar et al. 2011). All chimeric tags were removed and finally obtained effective tags (Xu et al. 2019).

### Taxonomy classification and diversity analysis

The UPARSE pipeline (Edgar 2013) was used to cluster the effective tags of ≥ 97% similarity into operational taxonomic units (OTUs). The tag sequence, with the highest abundance in each cluster, was selected as a representative sequence which was then classified into organisms by RDP classifier (Version 2.2) based on UNITE database (https://unite.ut.ee/) with a naive Bayesian model. To reveal alpha diversity, we expanded the OTU table which was supplemented by several diversity indices involving Chao1 value, ACE value, Shannon index and Simpson index. Chao1, Simpson and all other alpha diversity indices were calculated in QIIME (Caporaso et al. 2010). OTU rarefaction curve and rank abundance curves were plotted in QIIME.

Principal coordinate analysis (PCoA), based on unweighted UniFrac metrics, was performed to reveal the dissimilar relationship of the fungal communities within the samples. The weighted and unweighted unifrac distance matrix was generated by QIIME. PCoA of unweighted unifrac distances were calculated and plotted in the Vegan package in R software (Wickham 2009; Oksanen et al. 2010). Pearson correlation analysis on reflecting the potential correlation relationship between physicochemical parameters and diversity, abundance of fungal communities was conducted in GraphPad Prism (Oksanen et al. 2010; Revelle and Revelle 2015). The correlation coefficient matrix was generated by two-tailed *p*-value statistics. Statistical analysis of alpha diversity was conducted in IBM SPSS software by one-way ANOVA (with Dunnett T3 post hoc test) and unpaired *t*-test (SPSS Inc., Chicago). CANOCO 4.5 was used to conduct Redundancy Analysis (CCA) for depicting the effect of environmental factors on the ordination of samples and their composition taxa.

Each OTU was assigned to a functional guild (e.g., parasite fungi, plant pathogenic fungi, and saprotrophic fungi) using the FUNGuild database (Nguyen et al. 2016) which is currently the largest database for assigning fungal genera to one of several functional guilds based on a community annotated database of fungal taxa with known or suspected ecological functions. FUNGuild assigns function based on matches at the genus and species levels along with a confidence level. We only considered probable and highly probable confidence score guild assignments (Xu et al. 2017).

### Quantification of fungal abundance

To quantify the ITS gene copy number of fungi in each sample, quantitative PCR measurement and statistical analysis were employed by step one plus real-time PCR system instrument (Applied Biosystems). Primer pair ITS3_KYO2F/ITS4R was used to detect the fungal ITS gene with annealing temperature of 62 °C. The 20 µl qPCR system contained the following reagents: 1 µl of DNA template (tenfold-diluted to avoid interference of humic acids), 10 µl of Premix (Fast Start Universal SYBR Green Master, Roche), and 0.4 µl of forward and reverse primer (10 µM) for fungal qPCR.

One positive ligated plasmid of pGEM-T easy vector with gene fragments from previously prepared PCR products was used to make the successive tenfold dilution series for generating standard curves for fungal qPCR. Copy numbers of standard plasmid dilution were calculated by first measuring the DNA concentration by Nanodrop and then applied into the equation: abundance of gene copy number/µl = (amount µ^−1^ × 6.022 × 10^23^)/(length × 1 × 10^9^ × 660). Results deviated unreasonably from values in the replicate groups were omitted and undetermined results were deleted. Final adjusted standard curve properties were as following: *r*^2^ = 0.9994, Eff% = 93.89%. Statistical analysis of Welch’s *t*-test was calculated in Vegan package (version 2.5.3) (Oksanen et al. 2010).

## Results

### Culturable fungal isolates

**Fig. 2:**
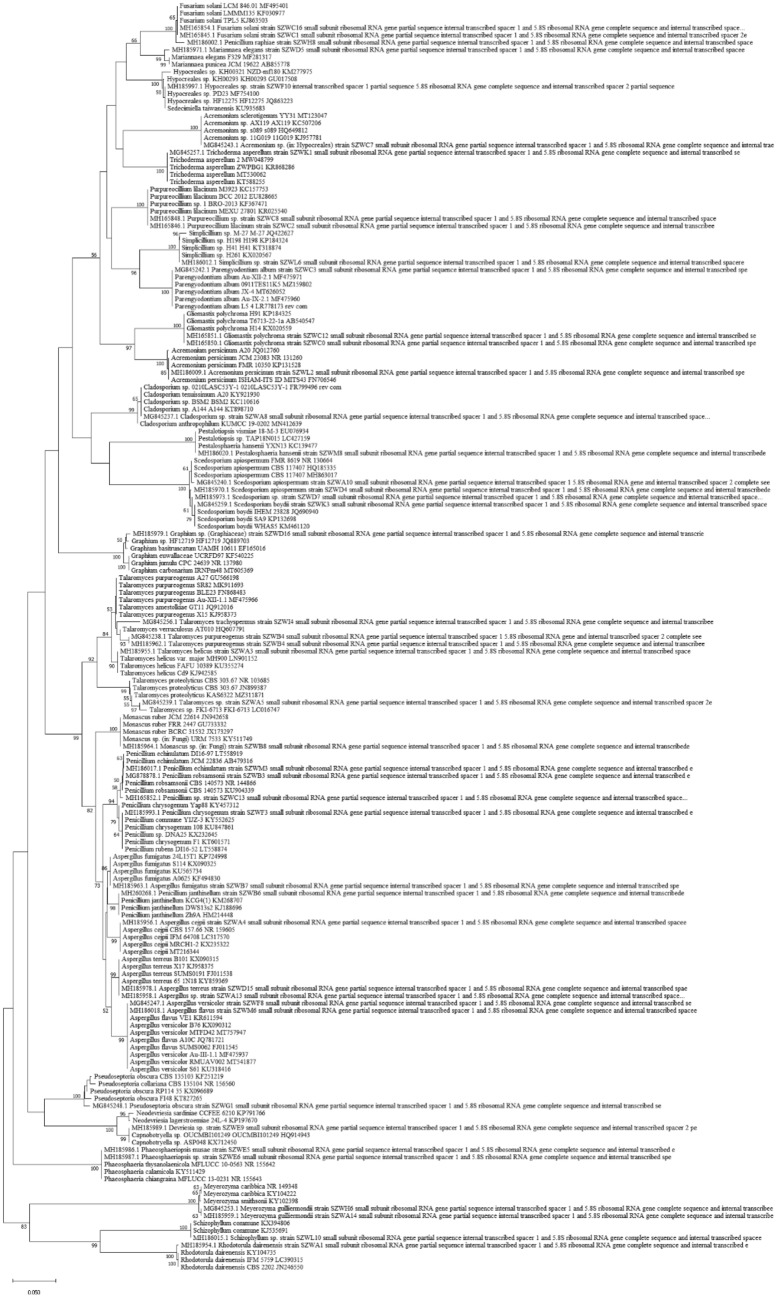
ITS tree of fungal culture isolates with reference sequence downloaded from NCBI cultured fungal database

A total of 115 fungi were isolated from the ten sediment samples. Then, these fungal isolates were selected for identification based on ITS sequencing. According to NCBI blast results, among the 115 isolates, most of them belonged to Ascomycota (112 isolates), only 3 isolates belonged to Basidiomycota. In the class level, Eurotiomycetes contributed the majority of isolates (46 isolates), followed by Sordariomycetes (40 isolates), 18 isolates belonging to Saccharomycetes and 8 isolates belonging to Dothideomycetes. Two Basidiomycota classes (Microbotryomycetes and Agaricomycetes), had 2 and 1 isolates, respectively. The phylogenic tree based on ITS2 of these isolated fungi is shown in Fig. 2 and their best matches in the NCBI database are also summarized in Table S1. In total, all identified fungi belonged to 23 genera of two phyla: *Acremonium*, *Aspergillus*, *Cladosporium*, *Capnobotryella, Parengyodontium*, *Fusarium*, *Hypocreales*, *Gliomastix*, *Graphium*, *Mariannaea*, *Meyerozyma*, *Monascus*, *Penicillium*, *Pestalotiopsis*, *Phaeosphaeria*, *Pseudoseptoria*, *Purpureocillium*, *Rhodotorula*, *Scedosporium*, *Schizophyllum*, *Simplicillium*, *Talaromyces* and *Trichoderma*. The fungal community was dominated by *Meyerozyma* and *Aspergillus*, with 18 and 17 strains, respectively, accounting for 30.4% of total fungi obtained, followed by *Penicillium* (14 isolates, 12.17%).

### Fungal community diversity and structure analysis

A total of 1,084,873 clean tags and 1750 OTUs were obtained from the sediment samples at ten sampling sites along the Shenzhen estuary. Species accumulation curves (Fig. S1) showed that the size of samples was adequate for subsequent data analysis. Good’s coverage (https://www.mothur.org/wiki/Coverage) scores were high across the compartments ranging from 0.998 to 0.999 indicating that the sequencing depth was adequate to dependably describe the fungal microbiome (Table 2).

**Table 2:** Comparison of the estimated operational taxonomic unit (OTU) richness, clean sequences, and diversity indexes of the fungal community structure in the sediments from the Shenzhen River Estuary

Sample	Clean sequences	Observed OTUs	Shannon index	Simpson index	Chao1 index	ACE index	Goods coverage
SZW1	124115	716	7.02	0.99	850	863	0.999
SZW2	103078	626	5.63	0.95	761	768	0.999
SZW3	107265	640	6.84	0.99	826	848	0.998
SZW4	102188	543	5.39	0.95	749	805	0.998
SZW5	111001	485	5.54	0.96	648	674	0.999
SZW7	121984	433	6.04	0.97	574	567	0.999
SZW8	91833	446	4.80	0.94	644	698	0.998
SZW9	106728	510	5.12	0.94	664	727	0.998
SZW10	110911	546	5.51	0.96	818	837	0.998
SZW11	105770	487	5.17	0.95	733	758	0.998

**Fig. 3:**
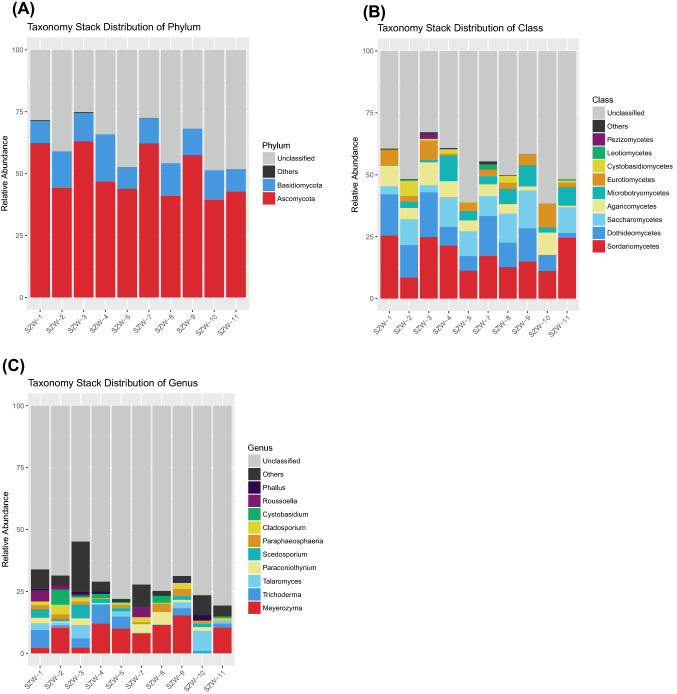
Relative abundance of the fungal phylum (**A**), class (**B**), and genus (**C**) recovered from ten sediment samples (SZW1-SZW-10) from the Shenzhen River Estuary by high-throughput Illumina sequencing

Of the fungal OTUs, 921 OTUs remained unclassified at the phylum level, and the other 829 OTUs were affiliated to seven phyla, 17 known classes, 51 orders, 104 families and 162 genera. Of these 1750 fungal OTUs, 37.3% (50.7% of all fungal reads) were assigned to the Ascomycota, followed by Basidiomycota (9.03% of OTUs, 11.7% of reads). Monoblepharidomycota, Chytridiomycota, Mucoromycota, Rozellomycota and Mortierellomycota were recovered in small proportions (1.09% of OTUs, 0.08% of reads). In the present study, at the phylum level, the composition of the fungal community in different stations was consistent and the relative abundance of Ascomycota was higher than other phylum (Fig. 3A).

Sequences from Ascomycota matched 12 known classes. The dominant ascomycete classes were the Sordariomycetes (17.37% of reads), Dothideomycetes (11.04% of reads), Saccharomycetes (8.21% of reads), Eurotiomycetes (4.15% of reads), Leotiomycetes (0.32% of reads) and Pezizomycetes (0.24% of reads). The most common basidiomycete classes were the Agaricomycetes (5.33% of reads), Microbotryomycetes (4.45% of reads), Cystobasidiomycetes (1.17% of reads), Tremellomycetes (0.24% of reads), Pezizomycetes (0.2% of reads) and Wallemiomycetes (0.2% of reads). Compare with other stations, Dothideomycetes in SZW-11 had the lowest relative abundance. Except for SZW-1, SZW-3 and SZW-10, Saccharomycetes widely distributed in other stations meanwhile had a high relative abundance (Fig. 3B).

There were 313 OTUs that could be assigned to 162 fungal genera. Among them, 12 genera (*Cladosporium*, *Cystobasidium*, *Meyerozyma*, *Paraconiothyrium*, *Paraphaeosphaeria*, *Penicillium*, *Pyrenochaetopsis*, *Roussoella*, *Scedosporium*, *Talaromyces*, *Trichoderma*, and *Gliocladium*) were present in all of the samples. *Meyerozyma* (3 OTUs) appeared with the most relative abundance of sequences (8.01%), followed by *Trichoderma* (5 OTUs, 3.08%), *Talaromyces* (25 OTUs, 2.38%), *Paraconiothyrium* (7 OTUs, 1.68%), *Cystobasidium* (3 OTUs, 1.67%), *Scedosporium* (8 OTUs, 1.61%), *Cladosporium* (2 OTUs, 1.39%), *Paraphaeosphaeria* (5 OTUs, 1.39%) and *Roussoella* (4 OTUs, 1.18%). Thirty genera with a very small proportion of reads (< 0.10%) were detected only in a single sample. As shown in Fig. 3C, *Meyerozyma* was the most abundant and dominant species in most of samples. However, SZW-1, SZW-3 and SZW-9 were a special case that the relative abundance of *Meyerozyma* was smaller than other fungi. Especially in SZW-10, *Meyerozyma* hardly existed (Table 3).

**Table 3:** Pearson correlation analysis between the physicochemical parameters and the abundance of fungal from the Shenzhen River Estuary

Var1^a^	Var2^b^	Correlation coefficient	*p*_value
ITS rRNA gene	Depth	− 0.57	0.09
ITS rRNA gene	pH	− 0.54	0.11
ITS rRNA gene	Salinity	− 0.34	0.33
ITS rRNA gene	Temperature	0.44	0.21
ITS rRNA gene	NO_2_^−^	− 0.20	0.57
ITS rRNA gene	NO_3_^−^	0.03	0.94
ITS rRNA gene	NH_4_^+^	0.72	0.02
ITS rRNA gene	TN^c^	0.50	0.14
ITS rRNA gene	IC^d^	0.62	0.06
ITS rRNA gene	TOC^e^	0.46	0.18
ITS rRNA gene	TC^f^	0.46	0.18

^a^Variable 1, the abundance of fungal in the ten samples were estimated using the fungal ITS rRNA gene copy numbers through qPCR

^b^Variable 2, physicochemical parameters

^c^TN (total nitrogen)

^d^IC (inorganic carbon)

^e^TOC (total organic carbon)

^f^TC (total carbon)

### Quantitative analysis of fungal ITS rRNA

Among the 10 samples, ITS gene abundance at site SZW-2 was higher than all the other sites (*t*-test), and sites SZW10 and SZW11 were lower compared to the other eight samples. Based on the Pearson correlation analysis between the physicochemical parameters and the abundance of fungal, ITS gene abundance was only positively correlated with NH_4_^+^ (*P* = 0.02, *R*^2^ = 0.52) (Fig. S2).

### Relationship of community properties and physicochemical parameters

The salinity of the Shenzhen estuary water gradually increased from the SZW-1 to the SZW-11. The salinity of the SZW-11 closest to the ocean was 28.07‰. The SZW-1 had the highest temperature. The NO_2_^−^ content was relatively low in each sediment, ranged from 0.01 to 0.039 mg N/kg. The NO_3_^−^ content ranged from 0.456 to 0.785 mg N/kg. In addition, NH_4_^+^ content in sediments near the estuary stations (e.g., SZW-1, SZW-2, SZW-3, SZW-4, SZW-5) was generally higher than the stations far away from the estuary. The TN was similar to NH_4_^+^, the IC content reached the highest value at the SZW-2 and SZW-3. However, the contents of TOC and TC were higher in the stations near the estuary than those far from the estuary (except SZW-5). In summary, the temperature and salinity of the water showed obvious gradients, while NO_2_^−^, NO_3_^−^, and IC had no gradient changes. Although TN, NH_4_^+^, TOC, and TC had no gradient changes, the difference between stations near the estuary and away from the estuary was obvious.

In terms of the relationship between alpha diversity indices and physicochemical parameters, Shannon indices of fungal communities were positively correlated with TOC, NH4^+^ and temperature, and negatively correlated with pH and Salinity by the Pearson correlation analysis, with statistical support (*p* < 0.05) (Table S3). At the same time, we found that the Shannon indices of sediments near the estuary (SZW-1, 2, 3, 4, 5 and 7) were greater than that of sediments far from the estuary but close to the ocean. This may indicate that the fungal diversity of sediments is more abundant near the estuary.

**Fig. 4:**
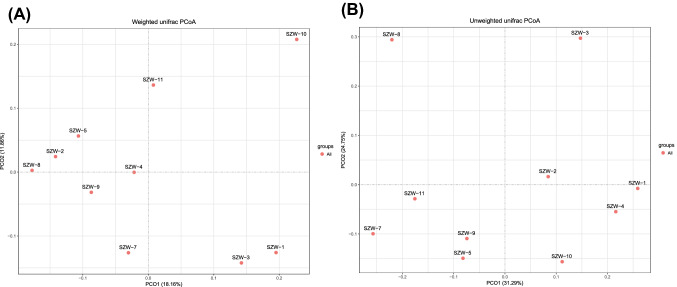
Principal Coordinate Analysis plots reflecting the dissimilar distance of fungal community among samples. The scatter plot is of principal coordinate 1 (PC1) vs. principal coordinate 2 (PC2). The percentages are the percentage of variation explained by the components

**Fig. 5:**
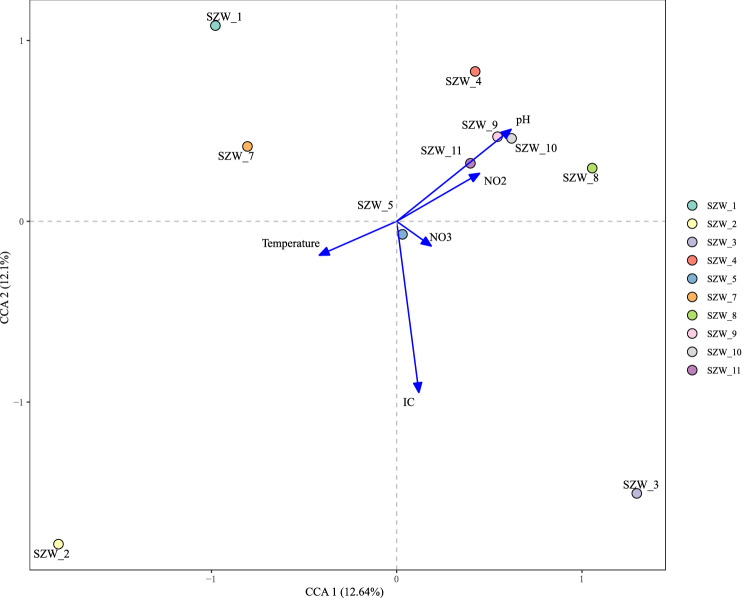
Plot redundancy analysis (CCA) integrating sampling stations and environmental factors from the ten sediment samples from the Shenzhen River Estuary

PCoA based on weighted Unifrac distance depicted variations in the fungal community at different sampling sites (Fig. 4A). The first two principal coordinates PC1 and PC2 of fungal PCoA explained 18.16% and 11.86% of the total variation. Unweighted Unifrac (Fig. 4B) showed the similar trend. The CCA results showed that the two ordination axes explained 12.64% and 12.1% variance for the fungal–environmental relationships in the Shenzhen River (Fig. 5), indicating that the abundant fungal assemblages were structured by different geochemical factors. The higher colinearity of physicochemical parameters was removed by Variance Inflation Factor (VIF). The results showed that pH and IC had more effect than other environmental factors.

### Functional guilds analysis

**Fig. 6:**
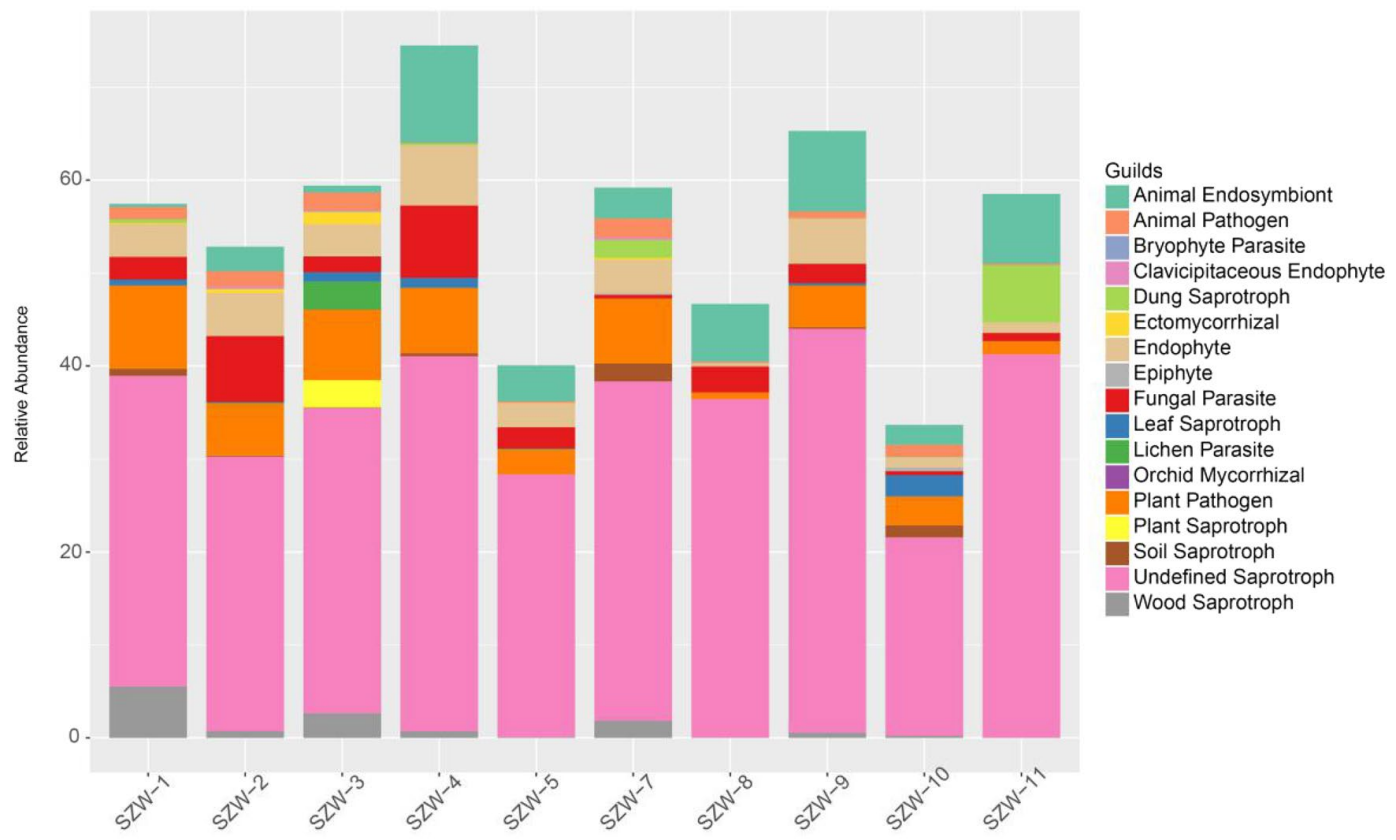
Relative abundance of fungal functional groups (guilds) based on the OTU annotation table with disturbance frequency level

FUNGuild database was used to classify the fungi in the present study by ecological guild. Most recovered sequences belonged to undefined saprotroph (28.09%), followed by animal endosymbiont (4.44%), endophyte (3%), plant-pathogen (1.85%), fungal parasite (1.33%), animal pathogen (0.93%), dung saprotroph (0.89%), leaf saprotroph (0.47%), wood saprotroph (0.33%), lichen parasite (0.32%), plant saprotroph (0.29%), ectomycorrhizal (0.21%), soil saprotroph (0.2%) and other fungi. Overall, most of the fungi in sediments of Shenzhen River Estuary were saprotroph (Fig. 6).

## Discussion

In this study, 46 of the 115 fungal isolates were selected for sequencing excluding some reduplicate strains. 46 identified fungi were successfully classified at the genus level based on ITS region sequences with relatives in the NCBI database. A total of 23 fungal genera were isolated, indicating rich fungal diversity in the samples. Most of them belong to *Meyerozyma*, which was a common yeast widely distributed in various environments, not only in the estuary environment but also in the deep-sea environment (for example, Sao Paulo Plateau) (Nagano et al. 2017). The second most dominant genera were *Penicillium* and *Aspergillus*, both of which were globally distributed as in Mandovi estuary (Gonsalves et al. 2012), Cabo Rojo Solar Salterns (Cantrell et al. 2006), the Gulf of Aqaba (Jaber et al. 2012) and the Changjiang River (Wu et al. 2013). The two were among 59 fungi isolates from the sediments of St. Helena Bay (Mouton et al. 2012). Investigation on culturable fungal communities in marine subsurface sediment cores in Suruga Bay found *Aspergillus* spp. as the most frequently detected fungal species (Nagano et al. 2016). *A. niger*, *A*. *flavus*, and *P*. *crysogenum* were some of the fungal isolates found from surface water of Indian rivers (Divya and Chouhan 2014). The studies also showed that the biological differences in these six waterways are mainly controlled by physical and chemical variables of samples, mainly pH and temperature. According to a previous study in Admiralty Bay, a total of 226 isolates were obtained, including 166 yeasts and 60 filamentous fungi (Wentzel et al. 2019). *Metschnikowia* is the most abundant yeast genus. For filamentous fungi, *Penicillium* and *Pseudogymnoascus* are the most abundant (Wentzel et al. 2019). According to the above results, we found that *Penicillium* and *Aspergillus* were widely distributed in estuaries and bay environments. *Acremonium, Aspergillus, Penicillium, Trichoderma* and *Rhodotorula* had been isolated in Shenzhen River Estuary and St. Helena Bay, but the diversity of cultivable fungi in Shenzhen River was higher. *Pichia* (also called *Meyerozyma*) was found in Suruga Bay (Japan), Shenzhen River Estuary (China), and Admiralty Bay (Antarctica). In general, the composition of cultivable fungal communities in different estuaries varied greatly.

In this study, we found that fungal assemblages residing in the Shenzhen River Estuary were assigned to 7 phyla and 51 orders. Our results are similar to a previous study on other estuary sediments (Li et al. 2016a), which was based on ITS1 metabarcoding data, reporting that Ascomycota followed by Basidiomycota was the most abundant phylum. Compared with culture approaches, we found that *Meyerozyma* was the most abundant genus in both Illumina sequencing and culture approaches. However, *Aspergillus* and *Penicillium* were relatively less abundant through Illumina sequencing, despite they have relatively large abundance in culture approaches. The relative abundance of Ascomycota was consistently and significantly higher than that of Basidiomycota both in culture method and Illumina sequencing approaches. Different from Shenzhen River Estuary, *Penicillium*, *Trichomerium*, *Nigrospora*, *Shiraia*, *Erythrobasidium*, *Mycosphaerella *et al*.* were reported to be the most common in the Bohai Bay (Li et al. 2016b). A pyrosequencing-based community study on fungi in freshwater lake sediments in the source area of the Yellow River showed that fungal community was dominated by Sordariomycetes, Leotiomycetes, Dothideomycetes, Pezizomycetes and Agaricomycetes (Tian et al. 2018). High-throughput sequencing analysis also suggests that there are obvious differences in the structure of fungal communities in different bays in China (Wahl et al. 2018). The ITS1 sequencing results showed that Agaricomycetes, Dothideomycetes, Saccharomycetes, and Sordariomycetes are dominant at the class level, while Agaricomycetes, Chytridiomycetes, Dothideomycetes, and Sordariomycetes are the dominant groups in ITS2 sequencing.

To describe fungal communities more accurately, we combine culture approaches with Illumina sequencing approaches (Table S2). Comparison of the fungal diversity obtained by ITS sequencing and cultivation method revealed that the two methods jointly found 23 fungal genera: *Acremonium, Aspergillus, Cladosporium, Capnobotryella, Parengyodontium, Fusarium, Hypocreales, Gliomastix, Graphium, Mariannaea, Meyerozyma, Monascus, Penicillium, Pestalotiopsis, Phaeosphaeria, Pseudoseptoria, Purpureocillium, Rhodotorula, Scedosporium, Schizophyllum, Simplicillium, Talaromyces* and *Trichoderma*, which indicated there were abundant marine fungi in the sedimental environment. However, the comparative analysis between the two methods makes us feel that only a small part of them had been isolated. Therefore, it is necessary to improve the laboratory separation technology and expand the scope of the marine research to obtain more new strains. Compare with culture approaches, Illumina sequencing approaches have the advantage that could generate huge amounts of DNA sequences. Rely on more sequences data, therefore, Illumina sequencing approaches can describe fungal community structure more accurately, and can overcome the shortcomings of the culture approaches very well. The traditional molecular approach (clone library analysis) indicated that culturable fungi were only a small fraction of the total fungi in deep-sea sediments (Nagano et al. 2010), and the clone library analysis represents a low throughput, costly and tedious method, providing only limited information of the diversity associated with the number of clones analyzed (Monchy et al. 2011). Therefore, based on high-throughput sequencing technology, we can gain a deeper understanding of the fungal community structure.

**Fig. 7:**
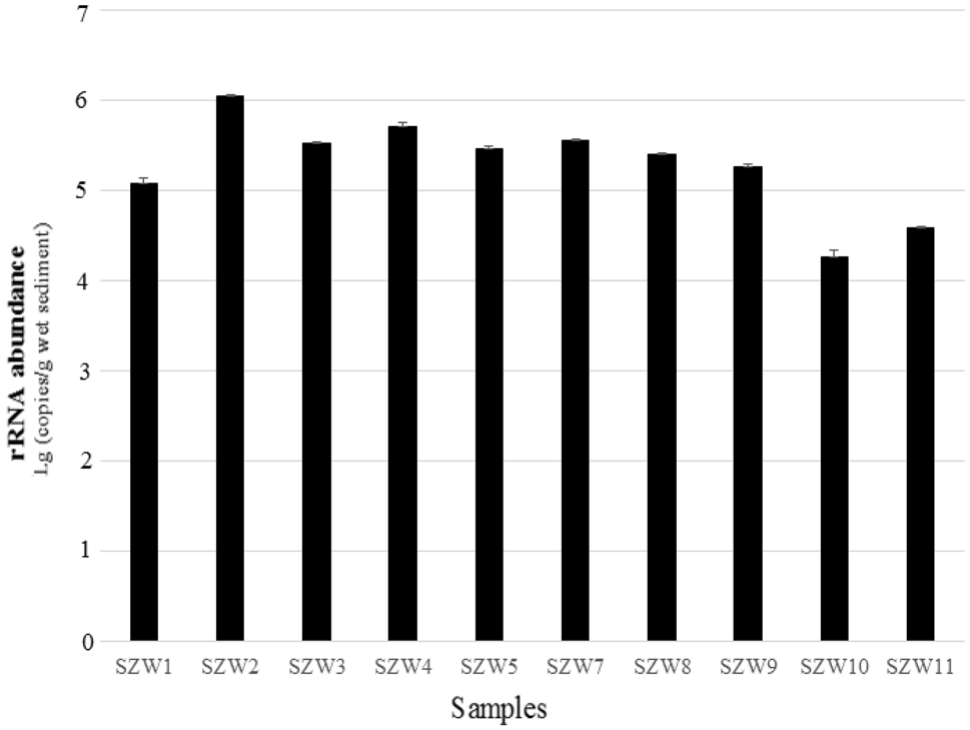
Abundance of the fungal ITS rRNA gene copies from ten sediment samples from Shenzhen River Estuary

The qPCR is a sensitive molecular tool to determine microbial abundance which is mostly used in conjunction with other assessments and measurements, such as community structure and chemical analyses (Jesser et al. 2015; Wang et al. 2018). The abundance of fungi in the ten samples was estimated using the fungal ITS rRNA gene copy numbers through qPCR. The fungal ITS rRNA gene copy numbers ranged from 1.85 ± 0.32 × 10^4^ to 1.10 ± 0.07 × 10^7^ copies/g (wet weight) (Fig. 7). Based on the Pearson correlation analysis between the physicochemical parameters and the abundance of fungi, ITS gene abundance was only positively correlated with NH_4_^+^ (*P* = 0.02), and not significantly correlated with other environmental factors (*P* > 0.05). This may suggest that NH_4_^+^ was a limiting factor affecting fungal growth in Shenzhen River Estuary. This finding is different from other reports. Because there are complex environmental factors in these three studying zone controlling the fungi community no only salinity but also C/N ratio and DO as well. That is why in Wang’s study (2018), fungal molecular abundance decreased with an increase of salinity, pH and DO (dissolved oxygen), They suggest that the low salinity and high nutrient status of SB (Shenzhen Bay) and PE (Pearl River Estuary) were favorable for the growth of planktonic fungi whereas the high salinity and low nutrient status of DB (Daya Bay) limited their growth and abundance.

Our results revealed that Ascomycota was the dominant fungi in Shenzhen River Estuary sediments. Fungal abundance and α-diversity were influenced by different environmental parameters. The Shannon index was significantly affected by salinity, NH4^+^and TN (*P* < 0.05), indicating that fungal diversity in sediments of Shenzhen River Estuary was affected by multiple factors. According to a previous research on fungal diversity in the Songhua River of China, organic matter, total nitrogen, ammonia nitrogen, and nitrate nitrogen are important influencing factors for fungal communities (Liu et al. 2015a, 2015b). Generally, the diversity or composition of fungi is considered to be affected by nitrogen utilization (Allison et al. 2007). Li et al. also proposed that salinity, total nitrogen (TN) and C/N significantly influenced the spatial distribution patterns of fungal communities (Li et al. 2016b). The results of coastal sites off the South China Sea (Pearl River estuary, Shenzhen Bay, and Daya Bay) showed that salinity and nitrate were the major factors driving the variations among fungal communities, and Ascomycota was positively correlated with salinity and negatively correlated with salinity and negative correlated with nitrate and nitrite (Wang et al. 2019). The above results indicate that nitrogen may be the most important factor affecting the fungal community structure (river or estuary). On the other hand, our study suggests that the Shannon index of sediment samples near the estuary (SZW-1, SZW-2, SZW-3, SZW-4, SZW-5 and SZW-7) was generally higher than that of sediment samples far from the estuary. This may indicate that the closer to the estuary, the higher the fungal diversity in the sediment. Furthermore, it is worth noting that we found the salinity of the sediment changed gradually from the estuary to the sea (low to high); the temperature gradually decreased; NH_4_^+^ gradually decreased; TN, IC, TC, and TOC also showed approximately gradient changes (higher near the estuary, lower away from estuary). This result was consistent with the characteristics of the estuary (Campbell and Kirchman 2013; Fu et al. 2015). Based on the CCA analysis, we found that salinity was significantly correlated with the majority of fungi in the genus level in Shenzhen River Estuary (a total of 17 genera). Salinity, in particular, has been implicated as a major factor regulating bacterial composition and diversity across many different habitats (Bernhard et al. 2005; Lozupone and Knight 2007). Our results also demonstrated that fungi were affected by salinity in the estuary environment, which is consistent with previous findings (Mohamed and Martiny 2011). In addition, TC and TOC were significantly correlated with 11 and 10 genera, respectively. *Trichoderma* was positively correlated with temperature, *Scedosporium* was significantly correlated with depth (negative), TN (positive), TOC (positive) and TC (positive); *Paraphaeosphaeria* was significantly correlated with NO_2_^−^; *Roussoella* was correlated with pH (Table S4). In general, although dominant fungi appear to be less affected by environmental factors, there was still a considerable proportion of fungal community which were significantly affected by the environment. The influence mechanism of environmental factors on fungal community needs to be further studied.

To better understand the ecological function of fungi, we conducted FUNGuild database analysis in this study. We found that saprotroph was the most abundant functional group across the ten samples. As we known, C/N ratio can roughly estimate terrigenous inputs of organic matter to aquatic environments, and saprotroph was the most abundant functional group across the ten samples further support the finding that fungi significantly affect the fate of the aquatic organic matter. Most of saprotrophs recovered were undefined (others were dung saprotrophs, leaf saprotrophs, plant saprotrophs, soil saprotrophs and wood saprotroph). A large number of saprotrophs found in Shenzhen River Estuary could promote the circulation of nutrients. They can produce more organic matter for other organisms to grow (Hunt et al. 1987). In addition, a large number of saprotrophic fungi were distributed in the Western English Channel, which may participate in the nutritional cycle (Taylor and Cunliffe 2016). Therefore, it was not surprising that the relatively high organic carbon content of sample SZW-3 resulted in high percentage of saprophytic fungi (41.29%). We speculate that organic matter of the samples may be from terrestrial input, and the impact of receiving terrestrial sources near the estuary may be comparatively great. The terrestrial materials from mega-river systems provide abundant dissolved or particulate organic and inorganic matter to coastal and estuarine regions, which can be carried offshore as far as the continental shelf (Mohamed and Martiny 2011), and nutrient inputs from land into estuaries have greatly increased through, predominantly, anthropogenic activities (Uncles et al. 2003; Statham 2012; Ram et al. 2014; Oviatt et al. 2017).

In summary, we combined the culture method with Illumina sequencing approaches to elaborate on the fungal community composition in the Shenzhen River Estuary. Meanwhile, we obtained a preliminary understanding of the ecological function of the fungal community by FUNGuild database analysis. Moreover, we analyzed the composition of fungal communities in sediments of different stations combined with environmental factors. Based on the Pearson correlation analysis between the physicochemical parameters and the abundance of fungal, ITS gene abundance was only positively correlated only with NH_4_^+^ (*P* = 0.02). Fungal community was significantly affected by pH and IC (*P* < 0.05), indicating that fungal structure in sediments of Shenzhen River Estuary was mainly affected by these two factors. As estuaries are transition zones between river and sea and are characterized by the mixing of freshwater, saline seawater, and sediment, it is speculated that the environmental factors influencing fungal distribution and community structure are multiple and complex. The present study may be able to enrich our understanding of the fungal ecological structure in the estuary environment.

## Supplementary Information

Below is the link to the electronic supplementary material.

Supplementary file1 (DOCX 211 KB)

## Data Availability

All data is available on NCBI.
