# Supplementary material for: Impact of environmental factors on diversity of fungi in sediments from the Shenzhen River Estuary
Source: Arch Microbiol. 2023 Feb 23;205(3):96. doi: 10.1007/s00203-023-03438-7 (PMC9950236; doi:10.1007/s00203-023-03438-7)
Supplement: Supplementary file 1 — Supplementary file1 (DOCX 211 KB) [file 203_2023_3438_MOESM1_ESM.docx]

**Impact of environmental factors on diversity of fungi in sediments from the** **Shenzhen River Estuary**

Wenzhen Lin^1,3,#^, Xin Liu^2,#^, Linfeng Gong^3^, Ruzhen Liu^3^, Minghuang Ling^3^, Chiming Guo^1^, Hongyan Meng^1^, Zhuhua Luo^3^, Xiaona Du^4^, Ying Guo^1,^*, Wei Xu^3,^*

^1^Fujian Key Laboratory of Subtropical Plant Physiology and Biochemistry, Fujian Institute of Subtropical Botany, Xiamen 361006, China

^2^School of Life Sciences, Anhui Agricultural University, Hefei 230036, China

^3^ Key Laboratory of Marine Biogenetic Resources, Third Institute of Oceanography, Ministry of Natural Resources, 178 Daxue Road, Xiamen 361005, China

^4^Sanquan College of Xinxiang Medical University, Xinxiang, Henan 453000, China

^#^ Wenzhen Lin and Xin Liu contributed equally.

***Corresponding author.**

**E-mail addresses: xwkhj@163.com (Wei Xu);** [**47402221@qq.com**](mailto:limeng848@szu.edu.cn) **(Ying Guo)**

**Running page head: Fungal diversity in estuary sediments**

**
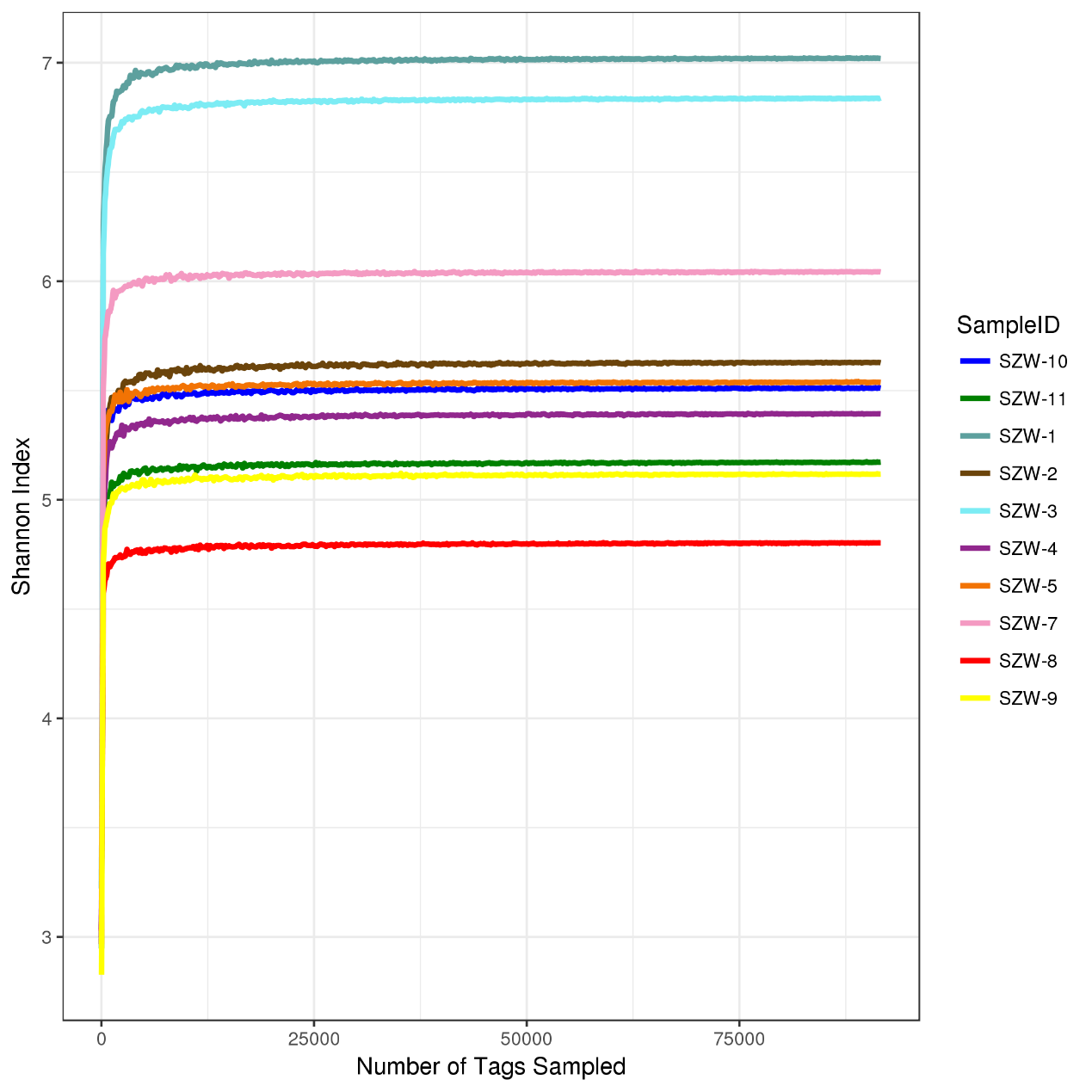
**

**Fig. S1** Shannon-Wiener curves for sediment samples from the Shenzhen River Estuary


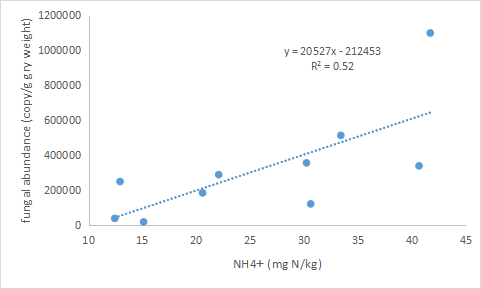


**Fig. S2** fungal abundance and its correlation with NH4^+^ from the Shenzhen River Estuary

**Table S1** Identiﬁcation of cultivable fungi from the Shenzhen River Estuary sediment samples on the basis of ITS gene sequences with ncbi BLAST hit and the phylogeny tree Evolutionary relationships of reference taxa.

| Isolate ID | GenBank accession number | Taxa identity based on phylogeny tree Evolutionary relationships from reference sequence in Fig S1 | Closest identiﬁed relative  (GenBank accession number) | Class/Phylum of closest identiﬁed relative | Coverage  (%) | Similarity (%) | Number of isolates |
| --- | --- | --- | --- | --- | --- | --- | --- |
| SZWA1 | MH185954 | *Rhodotorula dairenensis* | *Rhodotorula dairenensis* culture-collection CBS:4406(KY104735) | Microbotryomycetes /Basidiomycota | 100% | 100% | 2 |
| SZWA3 | MH185955 | *Talaromyces helicus* | *Talaromyces helicus* var. Strain MH900(LN901152) | Eurotiomycetes  /Ascomycota | 100% | 100% | 2 |
| SZWA4 | MH185956 | *Aspergillus cejpii* | *Aspergillus cejpii* (KF706672) | Eurotiomycetes  /Ascomycota | 100% | 100% | 6 |
| SZWA5 | MG845239 | *Talaromyces* sp*.* | *Talaromyces* sp*.* FKI-6713(LC016747) | Eurotiomycetes  /Ascomycota | 99% | 99% | 1 |
| SZWA8 | MG845237 | *Cladosporium* sp. | *Cladosporium* sp. BSM2(KC110616) | Dothideomycetes  / Ascomycota | 99% | 99% | 4 |
| SZWB4 | MG845238 | *Talaromyces purpureogenus* | *Talaromyces purpureogenus isolate X15*(KJ958373) | Eurotiomycetes  /Ascomycota | 99% | 99% | 4 |
| SZWA10 | MG845240 | *Scedosporium apiospermum* | *Scedosporium apiospermum* isolate G15G2C5(KY122867) | Sordariomycetes  /Ascomycota | 100% | 100% | 2 |
| SZWA13 | MH185958 | *Aspergillus terreus* | *Aspergillus* sp. YSN064(KT375568) | Eurotiomycetes  /Ascomycota | 99% | 99% | 5 |
| SZWA14 | MH185959 | *Meyerozyma* sp*.* | *Meyerozyma guilliermondii* culture-collection CBS:12037(KY104257) | Saccharomycetes  /Ascomycota | 100% | 100% | 16 |
| SZWB3 | MG878878 | *Penicillium robsamsonii* | *Penicillium robsamsonii* strain CBS 140574(KU904340) | Eurotiomycetes  /Ascomycota | 99% | 99% | 1 |
| SZWB4 | MH185962 | *Talaromyces purpureogenus* | *Talaromyces purpureogenus* isolate X15(KJ958373) | Eurotiomycetes  /Ascomycota | 100% | 100% | 3 |
| SZWB6 | MH260268 | *Penicillium janthinellum* | *Penicillium janthinellum* strain DYG1(1) (KM268707.1) | Eurotiomycetes  /Ascomycota | 100% | 100% | 1 |
| SZWB7 | MH185963 | *Aspergillus fumigatus* | *Aspergillus fumigatus* strain A0625 (KF494830) | Eurotiomycetes  /Ascomycota | 99% | 100% | 1 |
| SZWB8 | MH185964 | *Monascus* sp. | *Monascus* sp*.* Voucher URM7533(KY511749) | Eurotiomycetes  /Ascomycota | 100% | 100% | 2 |
| SZWC1 | MH165845 | *Fusarium solani* | *Fusarium* sp. Tm6-3(2) (AY433805) | Sordariomycetes  /Ascomycota | 99% | 99% | 1 |
| SZWC2 | MH165846 | *Purpureocillium lilacinum* | *Purpureocillium lilacinum* isolate 001JFC(KR025540) | Sordariomycetes  /Ascomycota | 99% | 100% | 1 |
| MG845242 | MG845242 | *Parengyodontium album* | *Parengyodontium album* isolate 0911TES11K5(LN808974) | Sordariomycetes  /Ascomycota | 99% | 99% | 7 |
| SZWC7 | MG845243 | *Acremonium* sp. | *Acremonium* sp. 11G019(KJ957781) | Sordariomycetes  /Ascomycota | 100% | 100% | 2 |
| SZWC8 | MH165848 | *Purpureocillium. lilacinum* | *Purpureocillium* sp. A5(KT239373) | Sordariomycetes  /Ascomycota | 100% | 100% | 1 |
| SZWC0 | MH165850 | *Gliomastix polychroma* | *Gliomastix polychroma* strain H14 (KX020559) | Sordariomycetes  /Ascomycota | 100% | 99% | 6 |
| SZWC12 | MH165851 | *Gliomastix polychroma* | *Gliomastix polychroma* strain H91(KP184325.1) | Sordariomycetes  /Ascomycota | 100% | 100% | 1 |
| SZWC13 | MH165852 | *Penicillium* sp*.* | *Penicillium* sp. LN0002(KM252957) | Eurotiomycetes  /Ascomycota | 100% | 100% | 10 |
| SZWC16 | MH165854 | *Fusarium. solani* | *Fusarium. solani* strain LMMM135(KF030977) | Sordariomycetes  /Ascomycota | 100% | 99% | 4 |
| SZWD4 | MH185970 | *Scedosporium* sp*.* | *Scedosporium. apiospermum* isolate C65F2(KY122857.1) | Sordariomycetes  /Ascomycota | 99% | 99% | 3 |
| SZWD5 | MH185971 | *Mariannaea elegans* | *Mariannaea elegans* var. Isolate: FKI-6644(AB855778) | Sordariomycetes  /Ascomycota | 99% | 97% | 1 |
| SZWD7 | MH185973 | *Scedosporium* sp. | *Scedosporium* sp. OTU053 AN-2016(KU556535) | Sordariomycetes  /Ascomycota | 100% | 100% | 1 |
| SZWD15 | MH185978 | *Aspergillus terreus* | *Aspergillus terreus* (KY926855) | Eurotiomycetes  /Ascomycota | 100% | 99% | 1 |
| SZWD16 | MH185979 | *Graphium* sp. | *Graphium* sp. HF12719(JQ889703) | Sordariomycetes  /Ascomycota | 100% | 99% | 1 |
| SZWE5 | MH185986 | *Phaeosphaeria/ Phaeosphaeriopsis* sp. | *Phaeosphaeriopsis musae* isolate A727(KU529841) | Dothideomycetes  /Ascomycota | 100% | 99% | 1 |
| SZWE6 | MH185987 | *Phaeosphaeria/ Phaeosphaeriopsis* sp*.* | *Phaeosphaeriopsis* sp*.* 014(KR012892) | Dothideomycetes  /Ascomycota | 99% | 99% | 1 |
| SZWE9 | MH185989 | *Capnobotryella/ Devriesia* sp. | *Devriesia* sp. W-2016b(KX237686) | Dothideomycetes  /Ascomycota | 99% | 99% | 1 |
| SZWF3 | MH185993 | *Penicillium* sp*.* | *Penicillium chrysogenum* isolate 108(KU847861) | Eurotiomycetes  / Ascomycota | 99% | 99% | 2 |
| SZWF8 | MG845247 | *Aspergillus versicolor/flavus* | *Aspergillus versicolor* isolate S61(KU318416) | Eurotiomycetes  /Ascomycota | 100% | 99% | 2 |
| SZWF10 | MH185997 | *Hypocreales* sp*.* | *Hypocreales* sp. HF12275(JQ863223) | Sordariomycetes  /Ascomycota | 99% | 99% | 1 |
| SZWG1 | MG845248 | *Pseudoseptoria* sp*.* | *Pseudoseptoria obscura* isolate RP114_35(KX096689) | Dothideomycetes  / Ascomycota | 99% | 99% | 1 |
| SZWH8 | MH186002 | *Fusarium/ Penicillium* sp. | *Penicillium raphiae* strain CBS126234(JN617673) | Eurotiomycetes  /Ascomycota | 100% | 99% | 1 |
| SZWH6 | MG845253 | *Meyerozyma* sp. | *Meyerozyma guilliermondii (Pichia gilliermondii)*ylx-8 (DQ657827) | Saccharomycetes  /Ascomycota | 100% | 100% | 2 |
| SZWI4 | MG845256 | *Talaromyces* sp. | *Talaromyces trachyspermus* strain: IFM 62335(LC317798) | Eurotiomycetes  /Ascomycota | 99% | 99% | 1 |
| SZWK1 | MG845257 | *Trichoderma asperellum* | *Trichoderma asperellum* strain ZWPBG1(KR868286) | Sordariomycetes  /Ascomycota | 99% | 99% | 1 |
| SZWK3 | MG845259 | *Scedosporium boydii* | *Scedosporium boydii* isolate SA9(KP132698) | Sordariomycetes  /Ascomycota | 100% | 99% | 1 |
| SZWL2 | MH186009 | *Acremonium persicinum* | *Acremonium persicinum* isolate 239 (MF351531) | Sordariomycetes  /Ascomycota | 99% | 99% | 1 |
| SZWL6 | MH186012 | *Simplicillium* sp*.* | *Simplicillium* sp. strain H261 (KX020567) | Sordariomycetes  /Ascomycota | 99% | 99% | 4 |
| SZWL10 | MH186015 | *Schizophyllum commune* | *Schizophyllum commune* voucher Fan & Guo 1303702(KX394806) | Agaricomycetes  /Basidiomycota | 99% | 99% | 1 |
| SZWM3 | MH186017 | *Penicillium echinulatum* | *Penicillium echinulatum* strain DI16-97(LT558919) | Eurotiomycetes  /Ascomycota | 100% | 99% | 1 |
| SZWM6 | MH186018 | *Aspergillus flavus/versicolor* | *Aspergillus flavus* strain VE1(KR611594) | Eurotiomycetes  /Ascomycota | 100% | 100% | 2 |
| SZWM8 | MH186020 | *Pestalotiopsis* sp. | *Pestalosphaeria hansenii* strain YXN13(KC139477) | Sordariomycetes  /Ascomycota | 99% | 100% | 1 |

Blue mark means the tax are a bit difference from NCBI blast hit and phylogeny tree.

**Table S2** Result comparison of culture-independent and culture-dependent methods

|  | Culture-independent method | Culture-dependent method |
| --- | --- | --- |
| **Ascomycota** |  |  |
| Eurotiomycetes | ● | ● |
| Dothideomycetes | ● | ● |
| Saccharomycetes | ● | ● |
| Sordariomycetes | ● | ● |
| Leotiomycetes | ● |  |
| Pezizomycetes | ● |  |
| **Basidiomycota** |  |  |
| Agaricomycetes | ● | ● |
| Microbotryomycetes | ● | ● |
| Cystobasidiomycetes | ● |  |
| Tremellomycetes | ● |  |
| Wallemiomycetes | ● |  |
| Exobasidiomycetes | ● |  |
| Ustilaginomycetes | ● |  |
| Pucciniomycetes |  |  |
| **Chytridiomycota** |  |  |
| Spizellomycetes | ● |  |
| Monoblepharidiomycetes |  |  |
| **Zygomycota** |  |  |
| Mucoromycetes | ● |  |
| **Rozellomycota** | ● |  |

“●”represents occur；

**Table S3** Pearson correlation analysis between the physicochemical parameters of sediments and the alpha diversity indices (Shannon index) of fungal communities from the Shenzhen River Estuary

| Var1^a^ | Var2^b^ | cor^c^ | p_value |
| --- | --- | --- | --- |
| Shannon | pH | -0.678 | 0.031 |
| Shannon | Salinity | -0.75 | 0.013 |
| Shannon | Tempature | 0.679 | 0.031 |
| Shannon | NO_2_^-^ | -0.666 | 0.035 |
| Shannon | NH_4_^+^ | 0.639 | 0.047 |
| Shannon | TN | 0.7 | 0.024 |
| Shannon | TOC | 0.731 | 0.016 |
| Shannon | TC | 0.73 | 0.017 |

a: variable 1, alpha diversity indices of fungal communities from the Shenzhen River Estuary; b: variable 2, physicochemical parameters; c: correlation coefficient;

e: IC (inorganic carbon);

**Table S4** Pearson correlation analysis between the physicochemical parameters of sediments and the fungi in sediments from the Shenzhen River Estuary.

| Var1^a^ | Var2^b^ | cor^c^ | p_value |
| --- | --- | --- | --- |
| Scedosporium | Depth | -0.6362 | 0.0480 |
| Gliocladium | Depth | -0.6930 | 0.0263 |
| Paecilomyces | Depth | -0.6980 | 0.0248 |
| Pleurostoma | Depth | -0.6534 | 0.0405 |
| Roussoella | pH | -0.6342 | 0.0489 |
| Tolypocladium | pH | -0.8292 | 0.0030 |
| Ganoderma | Salinity | -0.7565 | 0.0113 |
| Trematosphaeria | Salinity | -0.8303 | 0.0029 |
| Purpureocillium | Salinity | -0.8102 | 0.0045 |
| Trechispora | Salinity | -0.7963 | 0.0058 |
| Penicillium | Salinity | -0.8528 | 0.0017 |
| Fusarium | Salinity | -0.7966 | 0.0058 |
| Gliocladium | Salinity | -0.9439 | 0.0000 |
| Paecilomyces | Salinity | -0.8665 | 0.0012 |
| Cytospora | Salinity | -0.8157 | 0.0040 |
| Pleurostoma | Salinity | -0.8476 | 0.0020 |
| Marchandiomyces | Salinity | -0.7893 | 0.0066 |
| Saccharomyces | Salinity | -0.7879 | 0.0068 |
| Xylaria | Salinity | -0.8076 | 0.0047 |
| Apiotrichum | Salinity | -0.8056 | 0.0049 |
| Coniella | Salinity | -0.7886 | 0.0067 |
| Clathrus | Salinity | -0.7946 | 0.0060 |
| Geastrum | Salinity | -0.8090 | 0.0046 |
| Trichoderma | Tempature | 0.6675 | 0.0350 |
| Paecilomyces | Tempature | 0.6390 | 0.0467 |
| Paraphaeosphaeria | NO2- | 0.6789 | 0.0309 |
| Thermoascus | NO2- | 0.6436 | 0.0447 |
| Byssochlamys | NO2- | 0.6450 | 0.0440 |
| Schizophyllum | NO2- | 0.6492 | 0.0422 |
| Myxospora | NO_3_^-^- | 0.6829 | 0.0295 |
| Dictyophora | NO_3_^-^- | 0.7366 | 0.0151 |
| Exophiala | NH_4_^+^ | 0.6950 | 0.0257 |
| Tolypocladium | NH_4_^+^ | 0.6690 | 0.0344 |
| Scedosporium | TN^d^ | 0.6732 | 0.0329 |
| Chaetomium | TN | -0.6379 | 0.0472 |
| Ganoderma | TN | 0.6448 | 0.0441 |
| Exophiala | TN | 0.6533 | 0.0405 |
| Paecilomyces | TN | 0.6540 | 0.0402 |
| Scedosporium | TOC^e^ | 0.6855 | 0.0287 |
| Pyrenochaetopsis | TOC | 0.6510 | 0.0415 |
| Scleroderma | TOC | 0.6907 | 0.0270 |
| Ganoderma | TOC | 0.6487 | 0.0424 |
| Exophiala | TOC | 0.7983 | 0.0056 |
| Stephanonectria | TOC | 0.6815 | 0.0300 |
| Kluyveromyces | TOC | 0.6505 | 0.0417 |
| Epicoccum | TOC | 0.7003 | 0.0241 |
| Palmiascoma | TOC | 0.6412 | 0.0457 |
| Acrostalagmus | TOC | 0.6337 | 0.0492 |
| Scedosporium | TC^f^ | 0.6853 | 0.0287 |
| Nectriopsis | TC | 0.6322 | 0.0499 |
| Pyrenochaetopsis | TC | 0.6518 | 0.0411 |
| Scleroderma | TC | 0.6915 | 0.0268 |
| Ganoderma | TC | 0.6477 | 0.0429 |
| Exophiala | TC | 0.7987 | 0.0056 |
| Stephanonectria | TC | 0.6821 | 0.0298 |
| Kluyveromyces | TC | 0.6517 | 0.0412 |
| Epicoccum | TC | 0.7009 | 0.0239 |
| Palmiascoma | TC | 0.6422 | 0.0453 |
| Acrostalagmus | TC | 0.6349 | 0.0486 |

a: variable 1, fungi genera; b: variable 2, physicochemical parameters; c: correlation coefficient; d: TN (total nitrogen); e: TOC (total organic carbon); f: TC (total carbon).
